# Estimation of Time-Dependent Reproduction Numbers for Porcine Reproductive and Respiratory Syndrome across Different Regions and Production Systems of the US

**DOI:** 10.3389/fvets.2017.00046

**Published:** 2017-04-05

**Authors:** Andréia G. Arruda, Moh A. Alkhamis, Kimberly VanderWaal, Robert B. Morrison, Andres M. Perez

**Affiliations:** ^1^Department of Veterinary Population Medicine, College of Veterinary Medicine, University of Minnesota, St Paul, MN, USA; ^2^Environment and Life Sciences Research Center, Kuwait Institute for Scientific Research, Kuwait City, Kuwait

**Keywords:** time-dependent reproductive number, surveillance, porcine reproductive and respiratory syndrome, space–time clusters, porcine reproductive and respiratory syndrome incidence

## Abstract

Porcine reproductive and respiratory syndrome (PRRS) is, arguably, the most impactful disease for the North American swine industry, due to its known considerable economic losses. The Swine Health Monitoring Project (SHMP) monitors and reports weekly new PRRS cases in 766 sow herds across the US. The time-dependent reproduction number (TD-R) is a measure of a pathogen’s transmissibility. It may serve to capture and report PRRS virus (PRRSV) spread at the regional and system levels. The primary objective of the study here was to estimate the TD-R values for PRRSV using regional and system-level PRRS data, and to contrast it with commonly used metrics of disease, such as incidence estimates and space–time clusters. The second objective was to test whether the estimated TD-Rs were homogenous across four US regions. Retrospective monthly incidence data (2009–2016) were available from the SHMP. The dataset was divided into four regions based on location of participants, and demographic and environmental features, namely, South East (North Carolina), Upper Midwest East (UME, Minnesota/Iowa), Upper Midwest West (Nebraska/South Dakota), and South (Oklahoma panhandle). Generation time distributions were fit to incidence data for each region, and used to calculate the TD-Rs. The Kruskal–Wallis test was used to determine whether the median TD-Rs differed across the four areas. Furthermore, we used a space–time permutation model to assess spatial–temporal patterns for the four regions. Results showed TD-Rs were right skewed with median values close to “1” across all regions, confirming that PRRS has an overall endemic nature. Variation in the TD-R patterns was noted across regions and production systems. Statistically significant periods of PRRSV spread (TD-R > 1) were identified for all regions except UME. A minimum of three space–time clusters were detected for all regions considering the time period examined herein; and their overlap with “spreader events” identified by the TD-R method varied according to region. TD-Rs may help to measure PRRS spread to understand, in quantitative terms, disease spread, and, ultimately, support the design, implementation, and monitoring of interventions aimed at mitigating the impact of PRRSV spread in the US.

## Introduction

Although porcine reproductive and respiratory syndrome (PRRS) is, arguably, one of the most important diseases of swine affecting the North American industry, aspects of its transmission within production systems and within regions are not completely understood (1). Even though PRRS is endemic in North America, recurrent emergence of new PRRS virus (PRRSV) strains results in an epidemiological dynamic that resembles an epidemic condition for the disease (2, 3). PRRSV epidemics impact the swine industry and commonly require prompt mobilization of resources for diagnostics (i.e., sequencing of the virus), thorough investigations to understand the origin of the emerging PRRSVs, and implementation of effective control measures.

Surveillance is an integral part of strategies for control and elimination of PRRSV. There are a number of surveillance activities currently in place in the US; however, because PRRS is not reportable, surveillance strategies vary dramatically according to factors such as region and production system. A few examples of such surveillance activities are ongoing monitoring in breeding herds and gilt development units, and passive surveillance triggered by clinical symptoms.

The concept of near real-time disease surveillance is important in the context of emerging PRRSV strains given that rapid identification of an epidemic (i.e., emergence of novel PRRSV strains) will likely result in a reduction of outbreak duration due to timely implementation of prevention and control measures to decrease virus spread within and across geographical regions.

In the absence of a regulatory framework, initiatives aimed at monitoring PRRS in North American swine farms are voluntary in nature. One example of an effort intended to coordinate surveillance efforts in the US at the national level is the Swine Health Monitoring Project (SHMP). The SHMP is a voluntary project that aims to monitor the incidence of PRRS; it currently enrolls approximately 42% of the US sow population distributed in 19 states in the country. Interpretation of collected data to participants and the swine industry currently focuses on incidence. Additionally, the number of new cases and spatial–temporal clustering have been previously investigated and reported to describe PRRS trends and to identify PRRS epidemics (4–6). However, the rate of new cases over time, referred to as incidence, serves as a proxy for risk but does not contribute as a metric for the epidemic progression or prediction of its evolution.

There are other methods, however, that could serve as proxy for disease progression and that have not been sufficiently explored in measuring PRRS transmissibility. The basic reproductive number (*R*_0_) refers to the average number of secondary infections caused by a primary case and is commonly used to characterize the transmissibility potential of a disease in a completely susceptible population (7). In contrast, the effective reproductive number (*R*_e_) can be used to characterize transmissibility once a certain proportion of the population has been infected and is resistant (immune) (8), which would be an example for the case of PRRS in the US. The time-dependent reproduction number (TD-R) is a measure of disease transmissibility that can be estimated over the course of disease progression (9). The TD-R has been particularly useful for monitoring epidemic trends, identifying “super-spreader events,” measuring progress of interventions over time and for providing parameters for mathematical models (e.g., models to test interventions) (10).

The overall hypothesis of this study was that PRRS transmissibility, as measured by the TD-R, would not differ between regions and swine production systems within the US. This result would indicate that epidemiological dynamics are somewhat synchronized across regions, either because of seasonal weather changes or high connectivity among regions due to animal movements, as opposed to each region experiencing distinct temporal dynamics. Thus, our primary objective was to estimate the TD-R values for PRRSV using regional and system-level PRRS data from across the US, and to contrast it to incidence estimates and commonly investigated space–time clusters. We hypothesized that the peaks on the TD-R, incidence, and the space–time clusters would overlap. Furthermore, the secondary objective was to test whether the estimated TD-R were homogenous across four US regions. For this objective, the hypothesis was that the TD-R would be homogenous across all regions. Ultimately, results presented here will contribute to support the design and implementation of strategies for PRRS surveillance and control in the US.

## Materials and Methods

### Data Source

Source of data for the study here was the SHMP, which includes a cohort of farms that voluntarily agree to share PRRS data weekly. The PRRS status captured in this dataset followed slightly modified guidelines described elsewhere (11). Briefly, status included categories 1, 2, 2fvi, 2vx, 3, and 4. Status 1 designates actively infected herds (in which pigs were shedding the virus), status 2 indicates stable herds (no shedding detected in weaned pigs after following certain sample size requirements); 2fvi and 2vx refer to herds that were using live-virus exposure or modified live-virus vaccination as control strategies, respectively; status 3 defines herds that were provisionally negative (negative gilts introduced into the herd and remained negative); and status 4 denotes herds that were seronegative.

**Figure 1:**
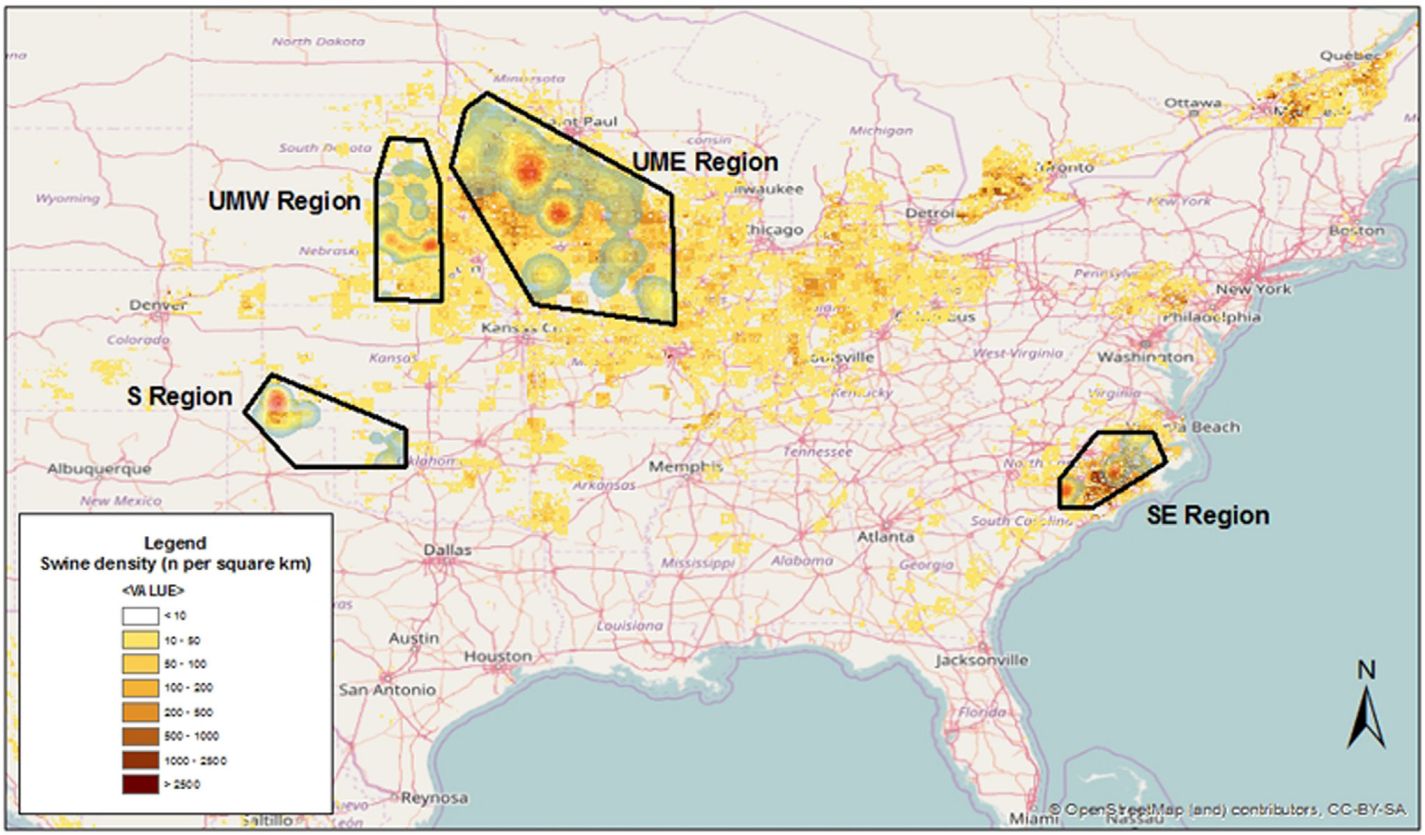
**Kernel smoothed density of swine sites used for this project overlaid on swine animal density as modeled by the FAO’s GeoNetwork data repository**.

Four areas across the US were chosen, including farms located in the states of North Carolina [South East (SE)], Oklahoma [South (S)], Minnesota/Iowa [Upper Midwest East (UME)], and Nebraska/South Dakota [Upper Midwest West (UMW)], and some neighboring locations (Figure 1). Those regions represented areas within the US characterized by high (SE and UME) and low (S and UMW) swine density, as reflected by the FAO’s GeoNetwork data repository for global livestock densities (Figure 1).

### Estimation of TD-R

The TD-R was estimated over time for farms participating in the SHMP project. Values of TD-R > 1 were interpreted as an indication that the number of cases would increase over time (propagating phase of an epidemic), whereas values of TD-R < 1 served as an indication that the epidemic was fading out (8). The estimation of reproductive numbers is commonly considered an indirect process, because the parameters needed (e.g., contact rate and probability of transmission given contact) are usually difficult, if not impossible, to estimate. Here, data available to compute the TD-R included weekly number of cases reported from July 2009 through March 2016.

Effective time-dependent reproductive numbers were estimated from observed incidence data using a likelihood-based procedure described elsewhere (8) and implemented through the R package “R0” in R v.3.2.3 (9). In summary, the TD-R was calculated based on averaging over all transmission networks compatible with the observed cases (9).

Firstly, incidence data were aggregated at the monthly level to reduce the prevalence of time intervals with 0 values in the time series. For months in which no cases were reported, the count of new cases was set to 1 with the assumption that at least one outbreak was missed, which is a reasonable assumption for PRRS, because sow herds have different levels of immunity due to variable management strategies, and these can impact detection of disease. Secondly, the generation time distribution that best fit the observed occurrence of cases was estimated. This refers to the time between detection of a primary case and detection of a secondary case (8), and in our case, we considered the time lag between consecutive reported outbreaks and estimated its mean and SD from the observed epidemic curve using a function in R (9). Thirdly, the number of secondary cases for each case was estimated by averaging over all transmission chains compatible with the epidemic curves during the course of epidemics. This was done in two steps (8):

First, the probability that a certain reported outbreak *i* (that occurred at a certain time) was infected by another reported outbreak *j* (occurring at a previous time) was calculated by *p_ij_* = *w*(*t_i_* − *t_j_*)*/*∑*_i≠k_w*(*t_i_* − *t_k_*); where *w* corresponds to the generation time distribution, and *t_i_* − *t_k_* corresponds to the difference in time of recording of outbreaks *i* and *j*.

Second, the TD-R for reported outbreak *j* was calculated by the sum over all outbreaks *i* weighted by the likelihood that outbreak *i* was infected by outbreak *j*: *R_j_* = ∑*_i_p_ij_*; and this was finally averaged considering all reported outbreaks with the same date of recording (9): 1*/N_t_*∑_{_*_tj_*_=t}_*R_j_*.

Confidence intervals (CIs) were obtained by simulation; and statistically significant periods of PRRS spreading were defined as periods [month(s)] for which the TD-R’s 95% CI did not include 1.

Time-dependent reproductive numbers were described separately for the four investigated geographical areas, as well as for each participating production system (20 systems represented by letters A–T). A production system was defined as two or more swine sites with a common owner or management structure. The Kruskal–Wallis test was used to determine whether the median TD-Rs differed across the four areas. Furthermore, the Dunn’s test of multiple comparisons (12) was applied, adjusting for multiple comparisons using the Bonferroni correction method. All statistical analyses were performed using STATA/IC version 14.1.

### Space–Time Permutation Model

Clustering of cases in space and time was explored using the permutation model of the scan statistic (13) implemented using the SaTScan™v.9.4.2 software (14). Briefly, the permutation model of the scan statistic compares the number of observed cases in any candidate cluster to the number of cases that would had been expected if the spatial and temporal location of all outbreaks were evenly distributed so that no space–time dependency occurred. The scan statistic has been proposed (15) as a surveillance tool to track clusters of disease, and it is especially useful because it does not require information on the background population at risk (16). Statistically significant clusters were declared when *P* < 0.05.

## Results

The number of outbreaks varied according to region, with SE and UME (North Carolina and Minnesota/Iowa), the most swine densely populated regions of the country, reporting the highest number of outbreaks over the 2009–2016 period (Table 1). A given swine site may have had more than one outbreak through the study period; the number of outbreak per site reporting an outbreak was higher for the S and UMW regions (1.76 and 2.21 outbreaks per site, respectively) when compared to SE and UME (1.44 and 1.48 outbreaks per site, respectively). Those two last areas, however, did contribute with a larger number of months of data (Table 1).

**Table 1:** **Basic regional descriptors and description of time-dependent reproduction number (TD-R) values calculated in the study for porcine reproductive and respiratory syndrome (PRRS) transmissibility between swine sites located across four different regions of the US**.

Region	*N* sites^a^	Period (months)^b^	*N* cases^c^	Median^d^	Mean (SD)^d^	Max [95% confidence interval (CI)]^d^	PRRS status before outbreak^e^ (% of sites reporting an outbreak)					
							1	2	2fvi	2vx	3	4
SE	72	81	104	0.99	1.14 (0.73)	5.42 (2.00, 9.00)	25.0	17.3	2.9	18.3	4.8	31.7
S	42	76	74	1.0	1.14 (0.54)	3.22 (1.00, 6.00)	0	4.0	0	85.1	0	10.8
UME	218	81	324	1.12	1.30 (0.68)	2.22 (0.45, 4.47)	5.4	8.8	39.3	20.5	7.7	18.3
UMW	38	76	84	1.002	1.10 (0.52)	2.80 (1.00, 5.00)	8.3	7.1	26.2	14.3	17.9	26.2

*SE, South East (North Carolina); S, South (Oklahoma); UME, Upper Midwest East (Minnesota/Iowa); UMW, Upper Midwest West (Nebraska/South Dakota)*.

*^a^Number of swine sites*.

*^b^Number of months the region contributed with data*.

*^c^Number of incident cases from 2009 to 2016*.

*^d^Median, mean (SD), and maximum (95% CI) for TD-R values calculated in this study*.

*^e^Status is according to AASV guidelines: status 1 designates actively infected herds (in which pigs were shedding the virus); status 2 indicates stable herds (no shedding detected in weaned pigs after following certain sample size requirements); 2fvi and 2vx refer to herds that were using live-virus exposure or modified live-virus vaccination as control strategies, respectively; status 3 defines herds that were provisionally negative (negative gilts introduced into the herd and remained negative); and status 4 denotes herds that were seronegative*.

The generation time distribution followed a lognormal distribution for the regions of SE (mean 1.30 months, SD: 1.26), S (mean: 1.09 months, SD: 1.01), UME (mean: 7.93 months, SD: 7.76) and UMW (mean months: 1.30, SD: 1.23).

**Figure 2:**
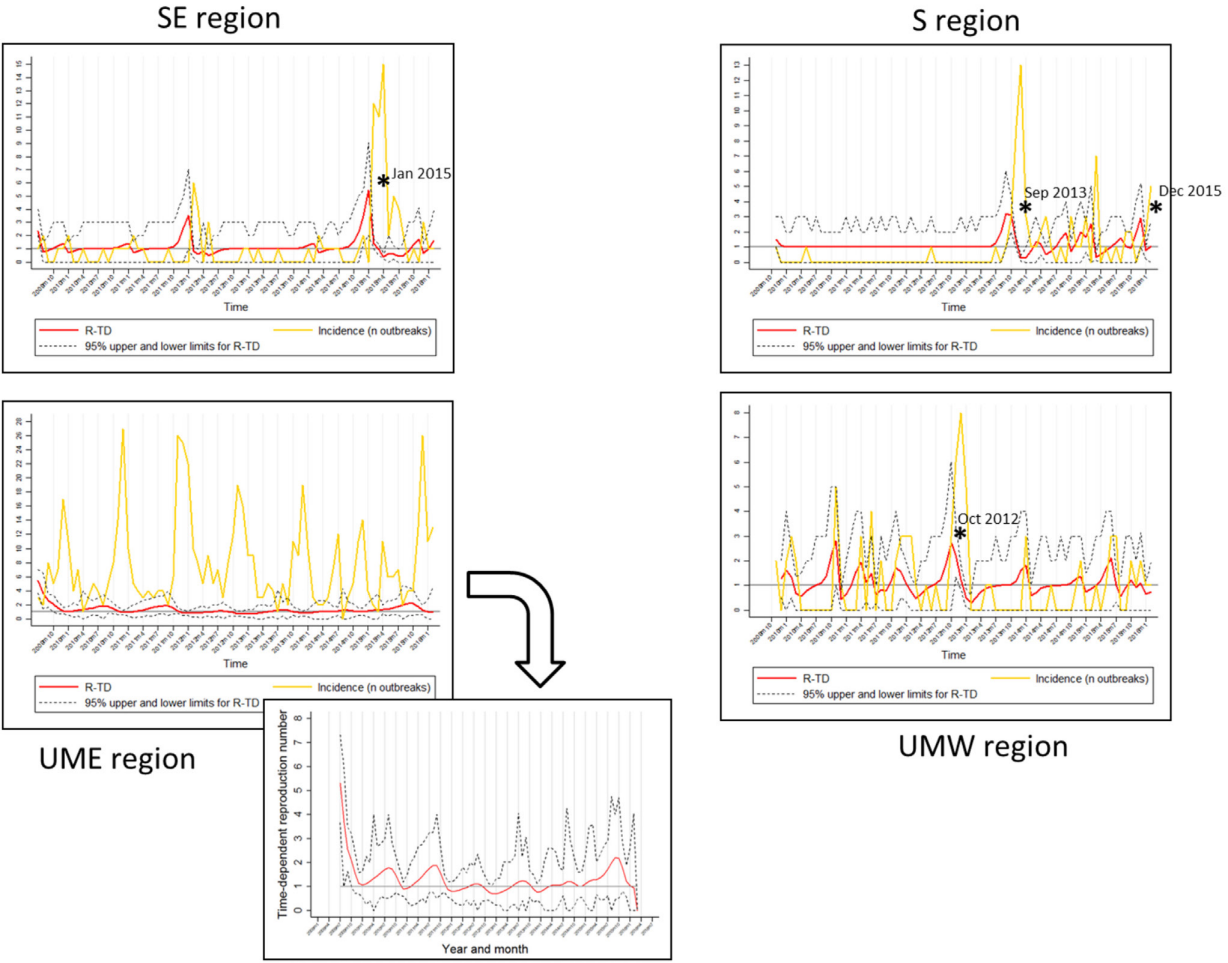
**Regional graphs showing time-dependent reproduction number (TD-R) and incidence for porcine reproductive and respiratory syndrome**. The *Y* axis corresponds to TD-R with 95% upper and lower confidence intervals (CIs) (red) and number of new cases (yellow; represented after replacing 0 counts with “1’s” as described in Section “Materials and Methods”). Stars (*) represent peaks on TD-R in which the 95% CI did not include 1; region Upper Midwest East (UME) has a zoom-out on the TD-R values to improve visualization.

The median and mean values for TD-R were similar across all regions and oscillated around 1.0, which is expected for endemic diseases. Interestingly, even though the mean and median values were close to 1 for all regions, incidence peaks and temporal variation in TD-R appeared remarkably different (Figure 2). A difference was observed in regards to the maximum number of TD-R values observed across regions; specifically, the TD-R was highest for SE, followed by S, UMW, and UME (Table 1). There were also remarkable differences in PRRS immune status classification for sites reporting outbreaks across the four regions (Table 1): of note; for the S region, the vast majority of sites reporting outbreaks were vaccinating the herd prior to the outbreak, which was not observed in such proportion for other regions. The SE region had a higher proportion of sites breaking that were classified as status 1 (active infection) when compared to sites that reported outbreaks from other regions (Table 1).

Between-region difference on median TD-R values was evident on the Kruskal–Wallis test, which showed that at least one of the regions had a different median (*P* = 0.03). Further *post hoc* pairwise comparison showed that the UME region was only statistically significant from the SE and UMW regions (Table 2).

**Table 2:** **Multiple pairwise comparisons for Kruskal–Wallis test using Dunn’s test of multiple comparisons, showing the estimate (*P*-value), with applied Bonferroni correction**.

Region	South East	South (S)	Upper Midwest East (UME)
S	−0.99 (0.96)	–	–
UME	−3.38 (0.002)*	−2.35 (0.0565)	–
Upper Midwest West	−0.42 (1.0)	0.56 (1.0)	2.91 (0.0107)

*Statistically significant difference.

It has been previously reported that PRRS has an evident seasonal pattern, showing high incidence during fall and winter (October through January), and low during spring and summer [February through September (5)]. Surprisingly, after stratifying the data by geographical region, there was no obvious visual indication of predictable yearly patterns for any of the regions besides Minnesota/Iowa (Figure 2).

The TD-R description showed variation according to geographical region, a phenomenon similar to the one previously described for the incidence estimate (Figure 2). The TD-R values showed statistically significant peaks before the incidence peaked for SE, S, and UMW (Figure 2). Interestingly, when comparing raw number of new cases or incidence with the TD-R estimates, all statistically significant peaks of TD-R (*P* < 0.05) preceded a meaningful increase in the number of cases (>2) for the regions of SE and S, showing the potential the tool has for early signaling outbreaks (Figure 2). The TD-R and the incidence peaks occurred at approximately the same time for the UMW region, and there were no statistically significant TD-R peaks for the region of MN/IA (Figure 2). For instances in which the TD-R peaks preceded the incidence peak, the lag time between peaks varied between 1 and 2 months. Likewise, indications that the epidemic was waning (TD-R < 1) occurred 2 months earlier than declines in incidence for SE and S (March 2015 versus May 2015 and November 2013 versus January 2014, respectively), and 1 month earlier for UMW.

**Figure 3:**
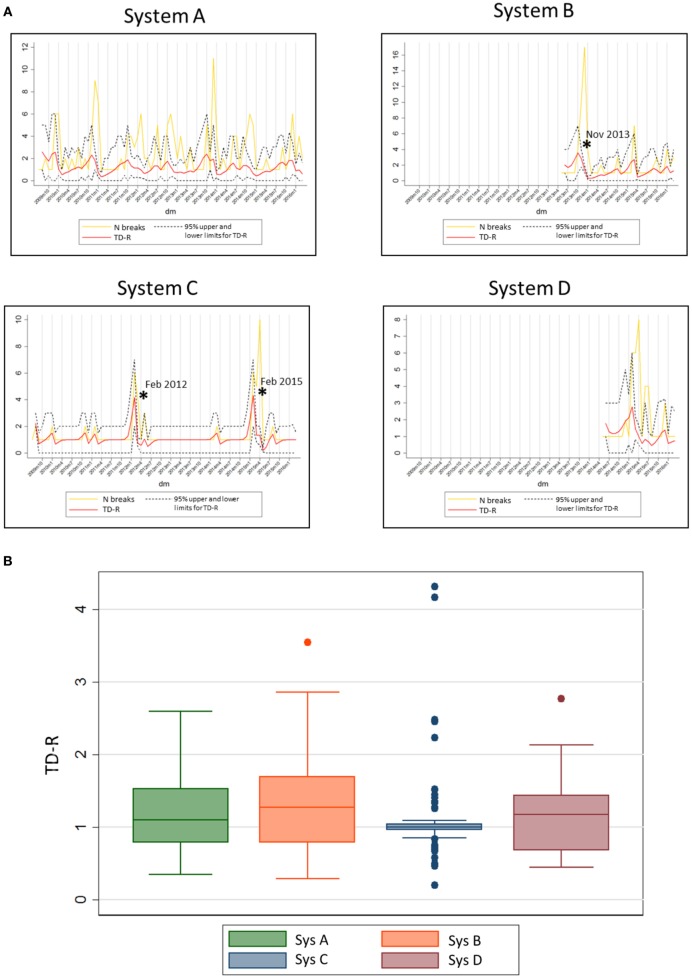
**(A)** System-specific graphs showing time-dependent reproduction number (TD-R) and incidence for porcine reproductive and respiratory syndrome. TD-R with 95% upper and lower confidence intervals (CIs) are shown in red, and incidence curves (after replacing 0 counts with “1’s” as described in Section “Materials and Methods”) are shown in yellow. Stars (*) represent peaks on TD-R in which the 95% CI did not include 1. **(B)** Box plot showing TD-R distribution for four different systems of the US.

Finally, the 20 systems examined contributed with a population at risk of on average 35 farms (min: 7, max: 83) per system. The average number of outbreaks per farm reporting at least one outbreak was 48.15 (min: 12, max: 189). Separate system-specific TD-R appeared to vary (Table 3; Figure 3), even for systems located within the same geographical region. Four systems were selected to illustrate differences between TD-R and incidence curves (systems A–D, Figure 3). Systems C and D, for example, were located within the same geographical region and showed one peak within the same time period (February 2015), even though the TD-R peak was not significant for system D. System A showed no significant peaks on TD-R, but it showed frequent increases in incidence; and system B showed a peak in TD-R not observed when farms are aggregated at the region level.

**Table 3:** **Time-dependent reproduction number summary estimates for each system enrolled in the SHMP project**.

System	*N* months^a^	Mean	Median	SD	Min	Max
A	80	1.21	1.09	0.53	0.34	2.60
B	34	1.36	1.28	0.78	0.29	3.55
C	80	1.12	1.00	0.61	0.20	4.31
D	22	1.19	1.18	0.58	0.45	2.77
E	80	1.09	1.00	0.41	0.32	2.10
F	80	1.04	1.00	0.31	0.33	3.00
G	80	1.19	1.00	0.63	0.32	3.62
H	80	1.04	1.00	0.31	0.25	2.42
I	80	1.04	1.00	0.29	0.25	2.42
J	80	1.15	1.00	0.62	0.28	3.97
K	80	1.02	1.00	0.23	0.33	2.93
L	80	1.03	1.00	0.32	0.33	2.99
M	53	1.07	1.00	0.58	0.24	5.00
N	80	1.01	1.00	0.22	0.5	2.00
O	80	1.07	1.00	0.47	0.33	3.52
P	80	1.05	1.00	0.37	0.38	2.80
Q	45	1.07	1.00	0.44	0.41	2.72
R	57	1.11	1.00	0.53	0.27	3.98
S	36	1.14	1.00	0.84	0.23	5.81
T	45	1.06	1.00	0.42	0.41	2.72

*^a^Number of months the systems are participating in the SHMP*.

The spatial–temporal model showed statistically significant clusters for all examined regions (Table 4). There were 3 clusters in space and time for the SE region, 3 clusters for the S region, 10 clusters for UME, and 4 clusters for UMW.

**Table 4:** **Super-spreader events and clusters found for the four regions by three different methods [time-dependent reproduction number (TD-R) estimation, purely temporal cluster detection, and spatial–temporal cluster detection]**.

Area^a^	TD-R^b^	Spatiotemporal cluster detection^c^	Radius of cluster (km)	O^d^	E^d^	*P*-value
SE	12/2014–01/2015	02/2015–07/2015	89	47	17.2	<0.001
		02/2012–07/2013	15	26	9.5	<0.001
		11/2010–12/2010	3.6	5	0.3	0.007
S	12/2015	08/2014–11/2014	42	10	1.9	<0.001
	10/2013	02/2014–05/2014	96	12	2.9	0.002
		11/2009–06/2012	7	4	0.1	0.007
UME		09/2015	4.3	22	1.7	<0.001
		07/2015–11/2015	44	35	9.7	<0.001
		01/2015–03/2015	22	12	0.6	<0.001
		04/2013–05/2013	44	15	0.7	<0.001
		09/2012	23	10	0.6	<0.001
		03/2012–05/2012	132	26	4.39	<0.001
		10/2011	3	15	1.52	<0.001
		07/2011–08/2011	65	13	0.7	<0.001
		05/2010–11/2010	94	47	13	<0.001
		01/2010–02/2010	7	31	6.5	<0.001
UMW	11/2012	06/2015–11/2015	42	15	1.9	<0.001
		05/2012–10/2012	31	10	1.6	<0.001
		06/2011	2	8	0.9	<0.001
		02/2010	28	14	3.9	0.01

*^a^Area 1 corresponds to North Carolina, area 2 corresponds to Oklahoma panhandle, area 3 corresponds to Minnesota/Iowa, and area 4 corresponds to Nebraska/South Dakota*.

*^b^Epidemic events as defined by TD-R (8); 95% confidence interval does not include 1*.

*^c^Spatial–temporal cluster detection using the spatial–temporal permutation model (14)*.

*^d^Observed (O) and expected (E) number of cases*.

## Discussion

The study here is the first to investigate and report the use of the time-dependent reproductive number for PRRS reporting purposes, and to contrast it with commonly used methods for describing PRRS epidemics (i.e., number of cases and spatial–temporal cluster detection). Strengths of this study include the availability of monthly PRRS incidence data from a large number of US swine herds spread across different geographical regions, as well as the inclusion of a large number of swine production systems.

Results support the observation that region-level insights cannot be provided by using data that are aggregated from large national projects. Furthermore, regional-level control and prevention strategies should not be made based on the assumption that PRRS transmission dynamics are the same across geographical regions of the same country. Stratification of data would be able to provide a better estimate on which control and prevention measures, if any, would work best and provide the best benefit for specific regions.

Comparison of PRRS transmissibility across regions and production systems has not been previously reported for PRRS, and the statistical differences among TD-R estimates between regions were somewhat surprising, given that it is commonly believed that all regions have similar PRRS transmissibility patterns. Some reasons that might explain the observed differences include climatic factors (e.g., temperature variation), demographic and biosecurity factors (e.g., presence of filtered farms), swine density, the presence of different production systems in the areas, and the potential introduction of PRRS strains in differing instances.

The commonly expected predictable yearly increase pattern for PRRS was not visually evident for all geographical regions across years, nor was the time periods in which PRRS was spreading (defined as the TD-R 95% CI did not include 1; Figure 2). Interestingly, even though both the SE and UME regions are known to have high swine density, the patterns of PRRS transmission between them were different (Figure 2). However, PRRS management strategies within these two regions are known to differ, which may partially explain the findings: first, the immunity status of swine sites is anecdotally observed to be different among areas. For example, among high swine dense areas (SE and UME), it is believed that a certain amount of herd immunity exists in the SE region compared to the UME because producers in the latter area are more willing to attempt PRRS elimination from herds. In contrast, producers from the SE region are commonly using vaccination or live-virus inoculation strategies to mitigate PRRS impact (SHMP data not shown). However, it was observed that, even when certain amount of underlying immunity existed for the SE region, spreading events still occurred. This is also anecdotally observed from field veterinarians and producers. Another difference between the areas might be the use of farm filtration as a preventive measure for PRRS outbreaks, with the SE area being characterized by lower frequency of filtered farms compared to the UME area. Data gather on these and other management and biosecurity factors for future projects might help elucidating regional differences described herein.

Porcine reproductive and respiratory syndrome epidemic events were recognized by the TD-R method for all regions except for the UME. These events possibly reflected the introduction of new or previously undetected PRRSV strains in the SE, S, and UMW areas (10, 17). Overall, the TD-R appeared to be particularly useful for areas where the occurrence of outbreaks is sporadic, perhaps resembling an epidemic nature. In such cases, the TD-R appeared to flag outbreaks of new strains earlier when compared to the crude increase in the number of cases (Figure 2), which could be valuable for near real-time disease surveillance in the context of commonly emerging PRRSV strains. The use of TD-R could aid in the rapid detection of these episodes, which, combined with communication and mobilization with key industry stakeholders, could result in faster control of disease in a region. For areas where PRRS can be characterized mostly as having endemic nature, the use of the TD-R might still be useful for signaling epidemic progression, characterizing transmissibility over time, and identifying the occurrence of “super-spreader” events.

The relatively large number of spatial–temporal clusters was not surprising. Analysis of data from a regional control project in Minnesota reported that, despite an overall decrease in PRRS incidence from 2012 to 2015, significant spatial–temporal clusters of disease incidence over 3-week periods and 3-km radii were found (5). The occurrence of spatiotemporal cluster did not overlap with the detection of peaks in TD-R as expected (Table 4) but usually was recognized later than the first. At times, these clusters were quite frequent and lasted for a long period of time, which raises the point to whether the alarms they may trigger would be of concern or not.

Finally, system-specific estimates of TD-R showed recognizable peaks for systems C and D (Figure 3). These peaks corresponded to a known incursion of an emerging strain for the area. In addition, for system C, there is empirical evidence that intense breaks occur every 3 years, which was evidenced by our analysis. System A was characterized by multiple outbreaks over time, even though statistically significant PRRS spread periods were not detected. Predictable yearly increase patterns were visually suggestive for this area, except for the most recent years. Finally, system B appeared to have had a large outbreak in the end of November of 2013, which once more is anecdotally thought to be due to the incursion of an emerging strain in the region. The authors hypothesize that the reason why no further considerable outbreaks were observed after these is a combination between control measures being taken after the epidemic event, and the existence of a certain level of immunity in the herd after infection. For future studies, collection of such information is important to allow for testing of these and other hypotheses. We also recognize that, at time of writing of this manuscript, peer-reviewed publications on this matter are largely lacking; therefore, it is challenging to compare our study results with previous work done in PRRS or any other swine infectious disease.

This study has some limitations. First, it is important to highlight that our source population corresponded to sow sites only and did not include growing pig sites. Even though growing pig sites are responsible for adding “infection pressure” at a regional level, one could argue that this population is somewhat distinct from the sow farm population in terms of disease management. Infection of sow herds results in more dramatic consequences due to the fact that pigs produced in such facilities are commonly transported to other sites, and therefore decisions in regards to disease prevention and control are markedly different between these distinct animal populations. On a similar note, our analysis included data from voluntary participants only. Therefore, results do not necessarily apply to the overall population of swine sites in the US. The impact of this issue is hard to predict and assess, given that the representativeness of participating producers is not well documented; thus, the authors recommend results to be taken with caution.

Second, underreporting could have affected results, especially for systems and regions that have underlying immunity for PRRS. To the knowledge of the authors, there are no published methods for TD-R calculations that can account for such issue, but the authors believe the underreporting to be constant in time, thus not dramatically affecting results. Another methodology-related issue is the fact that in our case the epidemic was not observed from the first case onward; with results in overestimation of the initial reproductive numbers (9). For this reason, the authors decided not to consider initial estimated TD-R numbers (first 4 months) when summarizing these data. Finally, although the prevalence of intervals with 0 values was not high, padding the time series, by replacing the 0 values with 1’s (a common practice in the time series analysis) resulted in a well-fitted distribution for the generation time, which subsequently increased the computation efficiency of the TD-R values. That said, this practice might have resulted in over or under estimation of the TD-R values in small regions with underreported outbreaks. However, as described above, the disease is endemic, and the assumption of at least one outbreak occurred in the small production region is biologically plausible in the context of epidemiology of PRRSV in the US. The authors also recognize that the CIs estimated using the time-dependent method could have been wide because few cases were observed at times. However, the authors are unaware of a method available (at time of publication) that would allow for better estimation of a transmission parameter in endemic settings.

In conclusion, this study showed the utility that TD-R estimates may have in monitoring and early signaling epidemics for PRRS, and its benefits will likely vary according to geographical region and production system. The TD-R is a promising complementary measure for incidence, because the latter is limited to measuring the amount of cases per unit of time but does not provide insights on the epidemic progression or effectiveness of control measures, which can be accomplished with the calculation of the former. The use of the TD-R may be complemented by other tools, such as, for example, the use of sequential Bayesian *R*_0_ for prediction of increases in the incidence as well as signaling the end of epidemics (18).

## Author Contributions

AA and MA formulated the main hypothesis of this study; AA was responsible for report and manuscript preparation and MA substantially helped with analysis. RM was responsible for acquisition of data. KV, AP, and RM helped with interpretation of results. All the authors contributed in critically revising the manuscript and approving its final version.

## Conflict of Interest Statement

The authors declare that the research was conducted in the absence of any commercial or financial relationships that could be construed as a potential conflict of interest.
